# Editorial: Mechanism and prevention of atrial fibrillation

**DOI:** 10.3389/fcvm.2022.1066542

**Published:** 2022-10-31

**Authors:** Guowei Li, Deirdre A. Lane, Potpara S. Tatjana, Yafei Li

**Affiliations:** ^1^Center for Clinical Epidemiology and Methodology, Guangdong Second Provincial General Hospital, Guangzhou, China; ^2^Liverpool Centre for Cardiovascular Science, University of Liverpool and Liverpool Heart and Chest Hospital, Liverpool, United Kingdom; ^3^School of Medicine, University of Belgrade, Belgrade, Serbia; ^4^Department of Epidemiology, College of Preventive Medicine, Army Medical University (Third Military Medical University), Chongqing, China

**Keywords:** atrial fibrillation, prevention, mechanism, prediction, basic science

## Introduction

Atrial fibrillation (AF) remains the most common sustained arrhythmia, with an estimated amount of more than 37 million patients globally (1). While AF is substantially related with increased risks of stroke and cardiovascular mortality, the prevention, diagnosis, treatment and management of AF continue to be suboptimal (2). With the emerging high-quality research and advanced progress on AF, this Research Topic aimed to collect state-of-art evidence and provide new insights into the mechanism and prevention of AF. Articles published in this Research Topic reported interesting findings and helped with our further knowledge of AF.

## Risk factors in relation to risk of AF

The survey study by Thirumal et al. explored the relationship between duration of lifetime occupational radiation exposure and risk of atrial arrhythmias, based on data from 1,478 cardiologists' responses in the US. They reported a significant association between elevated radiation exposure hours and increased risk of atrial arrhythmias, highlighting the preventive attention required when using fluoroscopy-assisted procedures for cardiologists. Another cohort study of Kim et al. conducted in Korea assessed the existence of diabetes mellitus in relation to stiff left atrial physiology in patients who had AF catheter ablation, by using a propensity score matching technique. Diabetes mellitus was found to significantly associate with increased risk of stiff left atrial physiology, while patients with stiff physiology were related with elevated risk of AF recurrence. A third investigation of Garcia et al., as a multicenter retrospective cohort study, reported that left circumflex artery obstruction was independently associated with increased risk of AF recurrence in patients who received catheter ablation and had no history of coronary artery disease (CAD), yielding a hazard ratio (HR) of 2.32 [95% confidence interval (CI): 1.36–3.98]. All these observational studies emphasized the clinical management to control the risk of arrhythmias.

There was another study of Musotto et al. depicting the morphological features of the left atrial appendage with thromboembolism risk under the condition of AF. This study demonstrated the active and passive contraction for hemodynamics in the left atrial appendage, and analyzed the hemodynamic role of AF in risk of thromboembolism. Results from this study provided some evidence of the mechanism of increased thromboembolism risk in AF by exploring hemodynamic parameters and anatomical phenotypes.

## Prediction of risk of AF

Three interesting studies in this Research Topic investigated prediction of AF. The first assessed the post-operative AF prediction of DNA methylation biomarkers in 221 patients receiving cardiac surgery, with an area under the curve (AUC) of 0.79 in the validation cohort for the prediction model. Findings from this study may provide some support in the combination of epigenomic and clinical information for predicting risk of post-operative AF (Fischer et al.).

Another prospective cohort study of Chen et al. explored the macrophage inflammatory protein-1 alpha (MIP-1α) for its link with left atrial remodeling in patients with AF. The significant relationship between a higher MIP-1α and larger volume of left atrial suggested the potential prediction of MIP-1α regarding the left atrial remodeling in AF.

Ortega-Martorell et al. used data from 18,518 patients admitted to intensive care unit (ICU) to build three prediction model of new episodes of AF: for the overall cohort, for the ventilated patients, and for the non-ventilated patients. The prediction models based on clinical information yielded good performance in predicting new episodes of AF, with an AUC of 0.84, 0.82, and 0.91 for the overall, ventilated and non-ventilated cohorts, respectively.

## Basic science in AF mechanism

One bioinformatic study of Ke et al. explored the competing endogenous RNAs (ceRNAs) by building the lncRNA-miRNA-mRNA network in AF, based on data from public databases. They found that based on the ceRNA theory, the network of LOC101928304/miR-490-3p/LRRC2 may be significantly related with AF, which may help deepen the understanding of AF pathogenesis and thus its prevention and treatment.

Wang and Tu summarized the progress of genetic and epigenetic research for ventricular arrhythmias in patients without structural heart disease. Unlike the other studies collected in this Research Topic that focused on atrial arrhythmias, their review generated an evidence map to aid in easy intake of most up-to-date regulatory mechanisms for ventricular arrhythmias including susceptibility genes, lncRNA, DNA methylation, histone modification, genomic imprinting, and 3D genomic architecture.

To summarize, this Research Topic collected interesting and novel research on the mechanism and prevention of AF. Results from these published studies expanded our understanding of pathogenesis, regulation, prevention and management of AF.

## Author contributions

GL and YL: conceived and designed the Editorial. GL: drafted the Editorial. DAL, PST, and YL: made revisions and provided support in the Editorial. All authors read and approved the submitted version.

## Conflict of interest

The authors declare that the research was conducted in the absence of any commercial or financial relationships that could be construed as a potential conflict of interest.

## Publisher's note

All claims expressed in this article are solely those of the authors and do not necessarily represent those of their affiliated organizations, or those of the publisher, the editors and the reviewers. Any product that may be evaluated in this article, or claim that may be made by its manufacturer, is not guaranteed or endorsed by the publisher.
